# Differential characteristics and outcomes of women and men undergoing Impella-supported high-risk PCI: IMPELLA-PL registry

**DOI:** 10.1016/j.clinsp.2025.100808

**Published:** 2025-12-05

**Authors:** Aleksandra Gąsecka, Arkadiusz Pietrasik, Jerzy Sacha, Tomasz Pawłowski, Marek Grygier, Michał Łomiak, Ewelina Błażejowska, Stanisław Bartuś, Łukasz Rzeszutko, Artur Pawlik, Pawel Kleczyński, Jacek Legutko, Janusz Kochman

**Affiliations:** a 1st Chair and Department of Cardiology, Medical University of Warsaw, Warsaw, Poland; b Department of Cardiology, University Hospital in Opole, Opole, Poland; c Faculty of Physical Education and Physiotherapy, Opole University of Technology, Opole, Poland; d National Medical Institute of the Internal Affairs ad Administration Ministry, Warsaw, Poland; e 1st Department of Cardiology, Poznan University of Medical Sciences, Poznan, Poland; f Department of Cardiology, University Hospital, Kraków, Poland; g Jagiellonian University Medical College, Institute of Cardiology, Department of Interventional Cardiology, John Paul II Hospital, Krakow, Poland

**Keywords:** High-risk percutaneous coronary interventions, Impella, Mechanical circulatory support, Men, Women, Mortality

## Abstract

**Background:**

Impella (Abiomed, Danvers, MA, USA) is a mechanical circulatory support device to prevent haemodynamic compromise in selected patients undergoing high-risk percutaneous coronary interventions.

**Objectives:**

Data on sex differences in the setting of Impella-assisted high-risk percutaneous coronary interventions are limited. This analysis compared the characteristics and outcomes of women and men supported with Impella CP during high-risk percutaneous coronary interventions.

**Methods:**

Consecutive patients enrolled in the IMPELLA-PL registry, undergoing high-risk percutaneous coronary interventions from January 2014 until December 2021, with 12-month follow-up until December 2022, were included in the analysis. The main endpoints included (i) In-hospital mortality, (ii) Periprocedural complications, (iii) 12-month mortality, and major adverse cardiovascular events following hospital discharge.

**Results:**

Among 253 patients analyzed, women (12.6 %) were older (75.2 ± 7.7 years vs. 70.1 ± 10.0, *p* = 0.006), had more left main disease (81.2 % vs. 61.1 %, *p* = 0.043), and higher Syntax Score (46.0 vs. 42.5, *p* = 0.038). The in-hospital mortality rate was comparable between groups (9.4 % vs. 8.1 %, *p* = 0.736). Women experienced fewer device-related complications (6.3 % vs. 20.8 %, *p* = 0.049), but more systemic complications (68.8 % vs. 35.7 %, *p* < 0.001). Women trended towards lower 12-month mortality (9.4 % vs. 19.4 %, *p* = 0.053), while post-discharge major adverse cardiovascular events (15.6 % vs. 23.5 %, *p* = 0.439) were comparable.

**Conclusions:**

Women and men undergoing Impella-assisted high-risk percutaneous coronary interventions have comparable in-hospital mortality rates, but different complication profiles. Following hospital discharge, the 12-month survival rate appears to be higher in women, despite more advanced age and a greater complexity of coronary artery disease.

## Highlights

• The authors compared outcomes of women and men supported with Impella CP during HR PCI.
• Women were older and had greater complexity of coronary artery disease.
• Women and men had comparable in-hospital mortality rates.
• Women experienced fewer device-related complications, but more systemic complications.
• Following discharge, the 12-month survival rate appears to be higher in women.

## Introduction

An increasing number of patients with coronary artery disease requires complex, high-risk revascularization procedures, posing a significant challenge for the Heart Teams worldwide. Although the exact definition of High-Risk Percutaneous Coronary Interventions (HR-PCI) remains a matter of debate, it generally encompasses various patient clinical, anatomical, and procedural characteristics, including acute coronary syndrome presentation, elderly age, extensive comorbidities, low left ventricle ejection fraction, three-vessel or left main disease, highly calcified lesions, complex bifurcation, or complex chronic total occlusion stenting.^1^ Multiple studies have demonstrated a so-called ‘risk-treatment paradox’ in patients, in which a higher risk of morbidity and mortality lowers the chance of receiving guideline-recommended care.^2^^,^^3^ This paradox is also observed in women and men undergoing HR-PCI.^4^ Women undergoing HR-PCI are older and burdened by more comorbidities and more challenging coronary anatomy compared to men.^5^ In addition, historical reports showed increased rates of PCI-related complications, such as coronary perforation and vascular access-site complications in women.^6^ These factors are likely responsible for the fact that women receive less revascularization and mechanical circulatory support.^7^ In contrast, recent studies have demonstrated that gender is not an independent predictor of adverse outcomes after HR-PCI after adjusting for other clinical features, such as left main lesion, last patent conduit, pulmonary hypertension, atrial fibrillation, anemia or renal failure.^8^ Hence, the previous reports indicating higher mortality in women undergoing HR PCI require re-evaluation.

Impella (Abiomed, Danvers, MA, USA) is a percutaneous mechanical circulatory support device recommended by the American College of Cardiology to prevent haemodynamic compromise in selected patients undergoing HR-PCI.^9^ Recent advancements in the Impella devices (e.g. introduction of Impella CP and Smart Assist System), increasing operator experience, and endorsement of standardized algorithms and institutional programs have contributed to improved outcomes after Impella-supported HR PCI procedures.^1^^,^^10^ Following the approval of Impella for clinical use in Europe in 2005, it has been adopted worldwide, with over 210,000 devices implanted to date.

Recently, the DanGer Shock showed that the routine use of Impella improved survival in patients with ST-segment elevation myocardial infarction-related cardiogenic shock, compared to standard care alone, despite the higher incidence of adverse events.^11^ The currently ongoing PROTECT IV trial is expected to provide the answer to whether the prophylactic Impella use might improve outcomes in the HR PCI setting as well.^12^

### Rationale

Despite the accumulating evidence on the efficacy and safety of Impella, data in various patient subpopulations, including men and women, are scarce. Hitherto, it has been shown that early mechanical circulatory support initiation may mitigate the excess mortality risk in female patients with cardiogenic shock.^13^^,^^14^ However, data on sex differences in the Impella-assisted HR-PCI setting are limited and controversial, showing comparable or higher rates of PCI-related complications in women, along with comparable or higher mortality rates.^15^, ^16^, ^17^, ^18^ To address this knowledge gap, the authors conducted a comparative analysis of the characteristics and outcomes of women and men enrolled in the IMPELLA-PL registry.

## Methods

### Study design

The IMPELLA-PL registry is a multicentre, investigator-initiated, retrospective registry including consecutive patients treated with Impella in the setting of cardiogenic shock and HR PCI in all Polish interventional cardiology centres that implanted at least 3 Impella devices between 2014 and December 2021. The study was conducted following the Strengthening the Reporting of Observational Studies in Epidemiology (STROBE) Statement. The 12-month follow-up ended in December 2022. All participating centres are public and most of them are academic. Since the introduction of the Impella device in Poland, it has been covered by the public healthcare system upon an individual's request. Hence, all patients included in the registry received Impella free of charge. The rationale and design of the registry, the list of participating centers along with the number of patients enrolled per center, and the efficacy and safety outcomes in cardiogenic shock and HR PCI patients were published previously.^19^, ^20^, ^21^

### Consent

The study protocol was approved by the Bioethical Committee of the Medical University of Warsaw (protocol number 1WR/5ABIOMED/2, version 1 from 4th January 2022).

### Patient selection

The study cohort consisted of hemodynamically stable patients with severe coronary artery disease who qualified for elective or urgent Impella-assisted HR-PCI by the local Heart Team. Patients in cardiogenic shock were excluded. Data regarding baseline characteristics, comorbidities, laboratory values, echocardiographic characteristics, angiographic and procedural characteristics, other cardiopulmonary support, the length of hospital stay, in-hospital and 12-month outcomes were collected based on the available medical records and inserted into dedicated, password-protected, web-based electronic case report forms, available via the dedicated study website (https://rejestrimpella.pl/). The electronic case report forms were designed and maintained by a dedicated IT specialist. At every site, 2 investigators were responsible for data collection (altogether 40 investigators) and follow-up. To ensure data completeness, the electronic case report forms did not allow the patient record to be saved unless all the required clinical data were filled in The follow-up regarding 12-month outcomes was conducted during the ambulatory visits or phone calls. In case the patient could not be contacted personally, the follow-up was completed based on the data from the national insurance database. Hence, there were no patients lost to follow-up.

### Clinical endpoints

The endpoints included in-hospital mortality, in-hospital major adverse cardiovascular events (exacerbation of heart failure, inflammatory complications, acute kidney injury, major bleeding as per operator judgement), device-related complications (access site bleeding, haemolysis, limb ischaemia, aortic injury), and 12-month outcomes, including mortality after discharge, recurrent hospitalization for HF, myocardial infarction, need for urgent repeated revascularization, heart transplantation, left ventricle assist device implantation, and stroke. The prespecified endpoint definitions were previously published.^19^

### Statistical analysis

Statistical analysis was performed using IBM SPSS Statistics, version 24.0. Categorical variables were reported using rates and proportions and compared using the Chi-Square test or Fisher's exact test for variables with fewer than five participants per category. Continuous variables were expressed as mean ± standard deviation or median (interquartile range) and compared using a *t*-test or a *U*-Mann-Whitney test, depending on the distribution. The long-term mortality rates were presented using Kaplan-Meier curves and compared using the log-rank test. All analyses were conducted by an independent statistician in a blinded manner. Statistical tests were two-sided, with a significance level of <0.05.

## Results

### Baseline characteristics

**Fig. 1:**
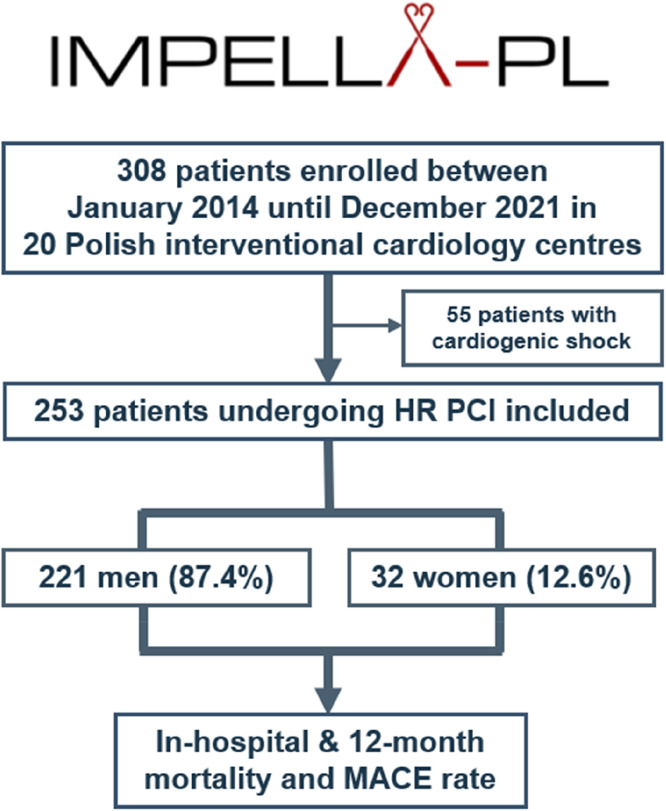
Study flow chart. Of 308 patients enrolled in the IMPELLA-PL registry, 253 underwent HR-PCI: 221 men (87.4 %) and 32 women (12.6 %). The HR PCI cohort consisted of haemodynamically stable patients with severe CAD who qualified for elective or urgent Impella-assisted HR PCI. Patients in cardiogenic shock were excluded. In-hospital and 12-month mortality, as well as 12-month MACE rates were evaluated in these patients. HR-PCI, High-Risk Percutaneous Coronary Interventions; MACE, Major Adverse Cardiovascular and Cerebrovascular Events.

The study flow chart is shown in Fig. 1. Of 308 patients enrolled in the IMPELLA-PL registry in 20 Polish interventional cardiology centers, 253 underwent HR-PCI, consisting of 221 men (87.4 %) and 32 women (12.6 %). The comparison of baseline characteristics between men and women is presented in Table 1. Compared to men, women were older (75.2 ± 7.7 vs. 70.1 ± 10.0, *p* = 0.006), had lower hemoglobin levels at admission (11.4 g/dL vs. 13.2 g/dL, *p* < 0.001), lower estimated glomerular filtration rate (58 vs. 63, *p* = 0.053), and a trend towards higher risk of post-PCI contrast nephropathy according to the Mehran Score (13 vs. 10 points, *p* = 0.051). In turn, women had lower N-terminal pro b-type natriuretic peptide (NT-proBNP) levels (1955 pg/mL vs. 4829 pg/mL, *p* = 0.022) and higher Left Ventricle Ejection Fraction (LVEF) (39.0 vs. 25.0, *p* < 0.001) compared to men. Acute coronary syndrome was the most common clinical presentation in women and men (56.3 % and 52.5 %, respectively). There were no differences in the rates of cardiovascular risk factors, comorbidities, and the median EuroSCORE between women and men.

**Table 1:** Baseline characteristics.

	Women (*n* = 32)	Men (*n* = 221)	p-value
**Age (years)**	75.2 ± 7.7	70.1 ± 10.0	0.006
**BMI (kg/m^2^)**	26.6 (24.1‒31.2)	27.2 (24.5‒30.5)	0.838
**Clinical presentation, n (%)**			0.850
ACS	18 (56.3)	116 (52.5)	
CCS	14 (43.8)	97 (43.9)	
**Comorbidities**			
Hypertension, n ( %)	27 (84.4)	172 (77.8)	0.539
Dyslipidemia, n ( %)	25 (78.1)	173 (78.3)	0.984
Diabetes mellitus, n ( %)	18 (56.3)	100 (45.2)	0.329
Prior myocardial infarction, n ( %)	16 (50.0)	116 (52.5)	0.792
Previous PCI, n ( %)	9 (28.1)	84 (38.0)	0.278
Previous CABG, n ( %)	1 (3.1)	26 (11.8)	0.219
Atrial fibrillation, n ( %)	8 (25.0)	67 (30.3)	0.538
Chronic heart failure, n ( %)	22 (68.8)	178 (80.5)	0.125
Previous stroke, n ( %)	2 (6.3)	22 (10.0)	0.749
Previous TIA, n ( %)	3 (9.4)	9 (4.1)	0.183
Chronic kidney disease, n ( %)	15 (46.9)	79 (35.7)	0.223
Dialysis, n ( %)	1 (3.1)	3 (1.4)	0.420
COPD, n ( %)	5 (15.6)	23 (10.4)	0.370
PAD, n ( %)	11 (34.4)	65 (29.4)	0.714
EuroSCORE, %	5.1 (2.7‒9.6)	5.0 (2.7‒9.1)	0.728
Mehran Risk Score, points	13 (8‒16)	10 (7‒14)	0.051
Cardiac arrest prior to admission, n ( %)	0 (0.0)	9 (4.1)	0.608
VF	0 (0.0)	4 (1.8)	1.000
VT	0 (0.0)	2 (0.9)	1.000
PEA	0 (0.0)	2 (0.9)	1.000
Asystole	0 (0.0)	1 (0.5)	1.000
ICD, n ( %)	2 (6.3)	41 (18.6)	0.139
Pacemaker	1 (3.1)	9 (4.1)	1.000
ICD	1 (3.1)	27 (12.2)	0.222
CRT	1 (3.1)	11 (5.0)	1.000
**Laboratory values**			
Haemoglobin (g/dL)	11.4 (9.8‒12.9)	13.2 (12.0‒14.4)	<0.001
Platelets ( × 10^9^/L)	242.0 (188.0‒277.0)	208.0 (167.0‒256.0)	0.089
eGFR (mL/min/1.73 m^2^)	58 (48‒67)	63 (49‒82)	0.053
CRP (mg/L)	10 (4‒26)	7 (2‒23)	0.449
NT-proBNP (pg/mL)	1955 (923‒3871)	4829 (1982‒10,383)	0.022
Troponin (ng/mL)	0.38 (0.03‒1.8)	0.31 (0.04‒3.5)	0.389
**Echocardiographic characteristics**			
LVEDD (mm)	52.0 (47.0‒57.8)	61.0 (54.8‒67.0)	<0.001
LA (mm)	42.0 (38.0‒45.0)	45.0 (42.0‒51.0)	<0.001
EF ( %)	39.0 (30.0‒50.0)	25.0 (20.0‒35.0)	<0.001
RV dysfunction, n ( %)	6 (18.8)	39 (17.6)	0.879
Mitral regurgitation grade 3‒4, n ( %)	5 (15.6)	38 (17.2)	1.000
Tricuspid regurgitation grade 3‒4, n ( %)	4 (12.5)	32 (14.5)	1.000
Severe aortic stenosis, n ( %)	0 (0.0)	3 (1.4)	1.000

Data presented as n ( %), mean ± standard deviation or median (interquartile range). T-test or *U*-Mann-Whitney test was used for continuous variables and Chi-Square test for categorical variables. ACS, Acute Coronary Syndrome; CCS, Chronic Coronary Syndrome; PCI, Percutaneous Coronary Intervention; BMI, Body Mass Index; CABG, Coronary Artery Bypass Graft; TIA, Transient Ischaemic Attack; COPD, Chronic Obstructive Pulmonary Disease; PAD, Peripheral Arterial Disease; EuroSCORE, European System for Cardiac Operative Risk Evaluation; VF, Ventricular Fibrillation; VT, Ventricular Tachycardia; PEA, Pulseless Electrical Activity; ICD, Implantable Cardioverter-Defibrillator; CRT, Cardiac Resynchronization Therapy; eGFR, Estimated Glomerular Filtration Rate; CRP, C-Reactive Protein; NT-proBNP, N-terminal pro B-type Natriuretic Peptide; LVEDD, Left Ventricular End-Diastolic Diameter; LA, Left Atrium; EF, Ejection Fraction; RV, Right Ventricle.

### Angiographic and procedural characteristics

Angiographic and procedural characteristics are shown in Table 2. There was a median of 3 vessels with significant stenosis in both groups, defined as > 50 % left main stem stenosis or >70 % stenosis in a coronary vessel > 2.5 mm in coronary angiography or 30 % to 70 % stenosis with fractional flow reserve ≤ 0.8. Nearly all patients presented with multivessel disease, with a higher percentage of women having multivessel disease, including left main (81.3 % vs. 61.1 %, *p* = 0.043). The median Syntax Score was higher in women (46.0 vs. 42.5, *p* = 0.038). All pre-planned lesions were successfully treated in 78.1 % of women and 83.7 % of men, with a higher rate of left main PCI in women (84.4 % vs. 67.0 %, *p* = 0.046). The contrast volume (190 vs. 250, *p* = 0.039) and radiation dose (952 vs. 1771, *p* = 0.010) were lower in women, compared to men.

**Table 2:** Angiographic and procedural characteristics.

	Women (*n* = 32)	Men (*n* = 221)	p-value
**Angiographic characteristics**			
Number of vessels with significant stenosis	3.0 (3.0‒4.0)	3.0 (3.0‒4.0)	0.517
Severe calcifications, n ( %)	18 (56.3)	122 (55.2)	0.911
Chronic total occlusions, n ( %)	15 (46.9)	122 (55.2)	0.377
In-stent restenosis, n ( %)	0 (0.0)	17 (7.7)	0.140
In-stent thrombosis, n ( %)	0 (0.0)	1 (0.5)	1.000
Intravascular imaging, n ( %)	11 (34.4)	93 (42.1)	0.448
IVUS	11 (34.4)	91 (41.2)	0.464
OCT	0 (0.0)	2 (0.9)	1.000
Functional assessment, n ( %)	1 (3.1)	8 (3.6)	1.000
Extent of the disease, n ( %)			
One-vessel	0 (0.0)	1 (0.5)	1.000
Multi-vessel (except for LM)	3 (9.4)	58 (26.2)	0.062
Multi-vessel (including LM)	26 (81.3)	135 (61.1)	0.043
Missing data	3 (9.4)	27 (12.2)	0.778
Syntax Score	46.0 (36.0‒69.0)	42.5 (30.9‒52.8)	0.038
**Procedural characteristics, n (%)**			
Vascular access for PCI, n ( %)			
Femoral artery	18 (56.3)	132 (59.7)	0.705
Radial artery	13 (40.6)	90 (40.7)	1.000
Brachial artery	1 (3.1)	6 (2.7)	1.000
Sheat size (French)	7 (6‒7)	7 (6‒7)	0.219
PCI via Impella sheath	6 (18.8)	39 (17.6)	0.879
Successful PCI in all lesions	25 (78.1)	185 (83.7)	0.515
Vessel treated:			
LM	27 (84.4)	148 (67.0)	0.046
LAD	29 (90.6)	169 (76.5)	0.070
CX	17 (53.1)	123 (55.7)	0.788
RCA	3 (9.4)	45 (20.4)	0.138
Contrast volume, mL	190 (120‒290)	250 (180‒350)	0.039
Radiation dose, mGy	952 (871‒2635)	1771 (518‒1863)	0.010
**Impella characteristics**			
Impella CP	31 (100.0)	221 (100.0)	1.000
Impella insertion, n ( %)			
Before PCI	29 (90.6)	178 (80.5)	0.303
During PCI	3 (9.4)	41 (18.6)	0.201
After PCI	0 (0.0)	0 (0.0)	1.000
Missing data	0 (0.0)	2 (0.9)	1.000
Explantation in cath lab, n ( %)	29 (90.6)	208 (94.1)	0.436
Duration of insertion (minutes)	25.0 (17.5‒41.0)	25.0 (15.0‒40.0)	0.934
Duration of support (hours)	16.3 (2.0‒100.0)	3.0 (2.0‒73.0)	0.358
Vascular access for Impella, n ( %)			
Femoral artery	29 (90.6)	178 (80.5)	0.303
Subclavian artery	1 (3.1)	13 (5.9)	1.000
Ultrasound-guided puncture	9 (28.1)	61 (27.6)	0.951
Surgical access	7 (21.9)	31 (14.0)	0.287
Contralateral safety access	2 (6.3 %)	20 (9.0 %)	1.000
**Other cardiopulmonary support**			
Use of catecholamines, n ( %)	4 (12.5)	43 (19.4)	0.482
Use of mechanical ventilation, n ( %)	1 (3.1)	9 (4.1)	1.000
Need for renal replacement therapy	1 (3.1)	3 (1.4)	0.420
Use of ECMO, n ( %)	1 (3.1)	4 (1.8)	0.560
Use of IABP, n ( %)	1 (3.1)	26 (11.8)	0.494
Intensive care stay, days	5.5 (2.5‒13.8)	3.0 (2.0‒8.0)	0.062
Hospital stay, days	12.0 (9.0‒17.0)	11.0 (7.0‒18.8)	0.601

Data presented as n ( %) and median (interquartile range). *t*-test or U-Mann-Whitney test was used for continuous variables and chi-square test for categorical variables. IVUS, Intravascular Ultrasound; OCT, Optical Coherence Tomography; PCI, Percutaneous Coronary Intervention; LM, Left Main coronary artery; LAD, Left Anterior Descending artery; CX, Circumflex Artery; RCA, Right Coronary Artery; cath lab, Catheterization laboratory; ECMO, Extracorporeal Membrane Oxygenation; IABP, Intra-Aortic Balloon Pump.

All patients were treated with Impella CP. In the majority of cases, Impella was inserted before PCI (90.6 % of women and 80.5 % of men) and was explanted in the catheterization laboratory (90.6 % of women and 94.1 % of men). The most common vascular access for Impella was the femoral artery (90.6 % of women and 80.5 % of men). The median duration of Impella insertion was 25 min in both groups. The median duration of support was longer in women, compared to men (16.3 h vs. 3.0 h), although the difference did not reach statistical significance. There were no differences in the use of catecholamines, mechanical ventilation, extracorporeal membrane oxygenation, and intra-aortic balloon pump between the groups. There was a trend towards longer stay in the intensive care unit in women, compared to men (5.5 days vs. 3.0 days, *p* = 0.062), with similar overall length of hospital stay in both groups.

### In-hospital and 12-month post-procedure outcomes

**Fig. 2:**
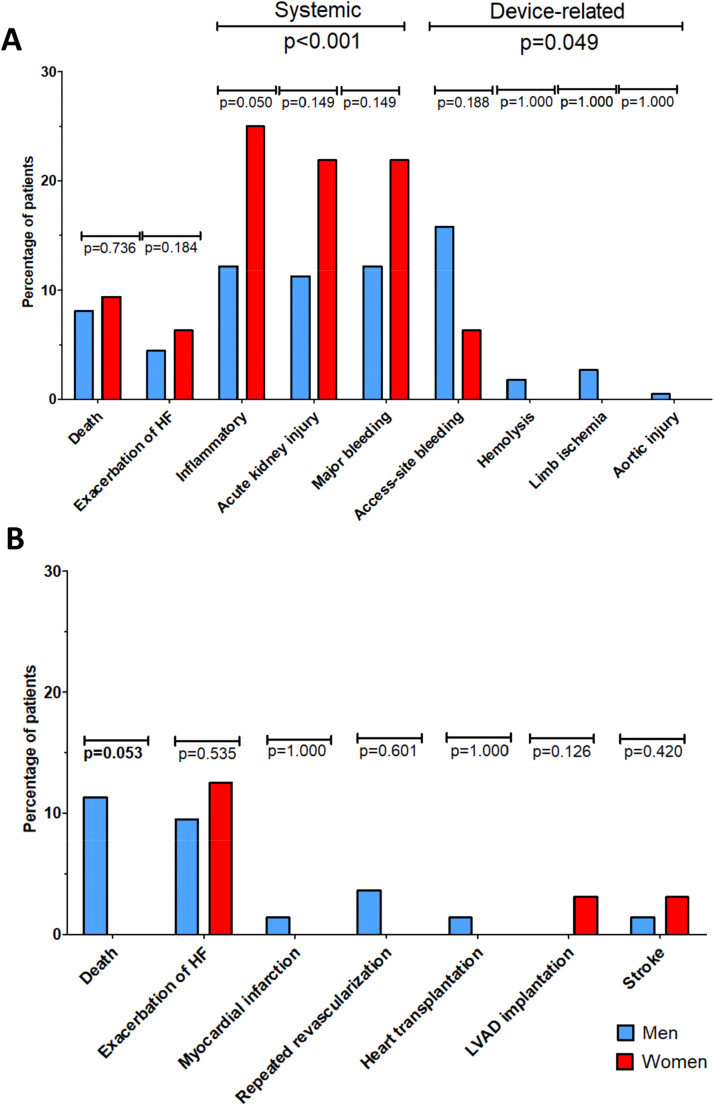
Comparison of in-hospital (A) and 12-month outcomes (B) in women and men undergoing Impella-supported high-risk percutaneous coronary interventions. Data presented as n ( %) and compared using chi-square test. Systemic complications are defined as inflammatory complications, acute kidney injury and major bleeding. Device-related complications are defined as access site bleeding, haemolysis, limb ischemia and aortic injury.

**Fig. 3:**
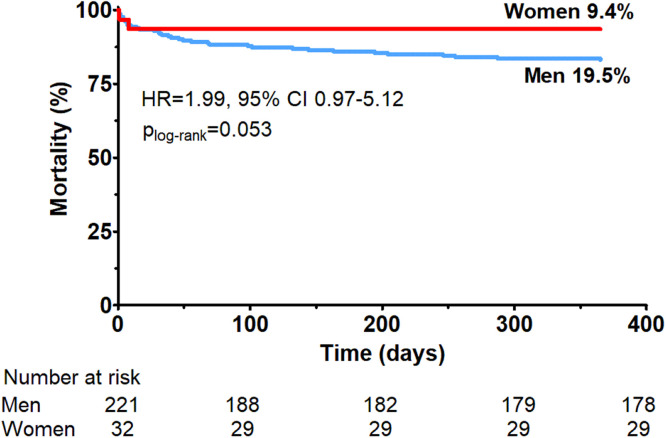
Kaplan-Meier survival curves of 12-month all-cause mortality post-discharge for men and women undergoing Impella-supported high-risk percutaneous coronary interventions, compared using the log-rank test. HR, Hazard Ratio; CI, Confidence Interval.

**Fig. 4:**
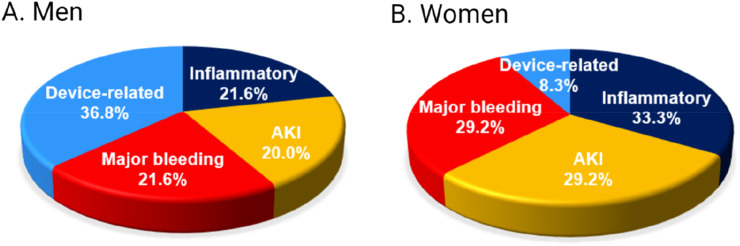
Complication profile in men and women undergoing Impella-supported high-risk percutaneous coronary interventions, shown as percentage of all (numerical) complications reported in each group. AKI, Acute Kidney Injury.

Comparison of in-hospital and 12-month outcomes is shown in Fig. 2 and Table 3. In-hospital mortality rate was comparable in women and men (9.4 % vs. 8.1 %). Women experienced more systemic complications, including inflammatory complications, acute kidney injury, and major bleeding, compared to men (68.8 % vs. 35.7 %, *p* < 0.001). In contrast, they experienced fewer device-related complications, including access site bleeding, hemolysis, limb ischemia, and aortic injury, compared to women (6.3 % vs. 20.8 %, *p* = 0.049). There was a trend towards a lower post-discharge mortality rate in women, compared to men, during the 12-month observation period (0.0 % vs. 11.3 %, *p* = 0.053). Furthermore, the overall 12-month mortality followed the same trend: 9.4 % in women vs. 19.4 % in men (*p* = 0.053) (Fig. 3). Comparison of the complication profile in men and women, shown as a percentage of all complications, is shown in Fig. 4.

**Table 3:** In-hospital and 12-month outcomes.

	Women (*n* = 32)	Men (*n* = 221)	p-value
**In-hospital outcomes, n (%)**			
Mortality	3 (9.4)	18 (8.1)	0.736
Exacerbation of heart failure	2 (6.3)	10 (4.5)	0.184
Systemic complications (number of patients)	14 (40.6)	62 (28.1)	0.097
Systemic complications (numeric)	22 (68.8)	79 (35.7)	<0.001
Inflammatory complications	8 (25.0)	27 (12.2)	0.050
Acute kidney injury	7 (21.9)	25 (11.3	0.149
Major bleeding	7 (21.9)	27 (12.2)	0.149
Device-related complications (number of patients)	2 (6.3)	39 (17.6)	0.126
Device-related complications (numeric)	2 (6.3)	46 (20.8)	0.049
Access site bleeding	2 (6.3)	35 (15.8)	0.188
Haemolysis	0 (0.0)	4 (1.8)	1.000
Limb ischaemia	0 (0.0)	6 (2.7)	1.000
Aortic injury	0 (0.0)	1 (0.5)	1.000
All complications (number of patients)	16 (50.0)	101 (45.7)	0.706
All complications (numeric)	24 (75.0)	125 (56.6)	0.048
**12-month outcomes, n (%)**			
Mortality after discharge	0 (0.0)	25 (11.3)	0.053
Hospitalization due to HF worsening	4 (12.5)	21 (9.5)	0.535
Myocardial infarction	0 (0.0)	3 (1.4)	1.000
Repeated revascularization	0 (0.0)	8 (3.6)	0.601
PCI	0 (0.0)	8 (3.6)	
CABG	0 (0.0)	0 (0.0)	
Heart transplantation	0 (0.0)	3 (1.4)	1.000
LVAD	1 (3.1)	0 (0.0)	0.126
Stroke	1 (3.1)	3 (1.4)	0.420
Total mortality	3 (9.4)	43 (19.4)	0.053

Data presented as n ( %). Chi-Square test for categorical variables. HF, Heart Failure; PCI, Percutaneous Coronary Intervention; CABG, Coronary Artery Bypass Graft; LVAD, Left Ventricle Assist Device.

## Discussion

The main findings of this study are that women and men undergoing Impella-assisted HR PCI have comparable in-hospital mortality, but different complication profiles, with more device-related complications in men and more systemic complications in women, and there is a trend towards lower 12-month mortality in women, despite more advanced age and higher complexity of coronary artery disease.

### Sex differences in the setting of Impella-assisted HR PCI

Data regarding sex differences in the setting of Impella-assisted HR PCI are limited and controversial. In the sub-analysis of the prospective, multicentre, observational PROTECT III study, including 1237 patients undergoing HR-PCI supported with Impella 2.5 or Impella CP, female patients were older, more often anemic, and had worse renal function, but higher LVEF compared to male patients,^7^ which is in line with these results. Female patients had a higher rate of immediate PCI-related coronary complications, but there were no sex differences regarding 90-day mortality and major adverse cardiovascular events (all-cause death, myocardial infarction, stroke/transient ischemic attack, and repeat revascularization). Similarly, in the subanalysis of the global Catheter-based Ventricular Assist Devices registry, which included 1053 patients who underwent HR-PCI using Impella 2.5 or Impella CP, women were older and had more comorbidities (diabetes, renal insufficiency, and peripheral vascular disease), compared to men.^16^ Following PCI, women had a higher rate of bleeding requiring transfusion, but there were no differences in 30-day mortality and major adverse cardiovascular events (stroke, MI, need for recurrent revascularization) between the groups. A propensity-score matching analysis of 4381 HR-PCI patients from the United States National Readmission Database also showed a higher rate of major bleeding in women, but a similar rate of vascular complications and in-hospital mortality.^18^ In a sub-analysis of the Impella Italian registry examining sex-dependent differences in clinical outcomes, women undergoing Impella-assisted HR-PCI had a higher burden of coronary anatomical complexity (left main disease and severely calcified lesions), received pre-PCI support less frequently, and required peri‑procedural resuscitation.^17^ The rates of in-hospital mortality and device-related complications were similar in both groups. However, women had higher 12-month mortality, compared to men, with a similar rate of 12-month major adverse cardiovascular events (heart failure rehospitalization, LVAD implantation, heart transplantation).

### Complications in women undergoing Impella-assisted HR PCI

Similar to previous studies, results from the registry population show that women undergoing Impella-assisted HR-PCI have more complications in general, which is driven by a higher number of inflammatory complications, acute kidney injury, and major bleeding. Inflammatory complications in the IMPELLA-PL registry were defined as infections accompanied by pain, fever, and/or leucocytosis, treated with antimicrobial agents, confirmed by the presence of positive culture test or strong clinical evidence despite negative cultures. The higher rate of inflammatory complications registered in women in the IMPELLA-PL registry might be due to the pro-inflammatory effect of estradiol, which leads to stronger immune responses than men experience, but better outcomes following bacterial infections.^22^^,^^23^ Hence, while women might have more pronounced inflammatory responses than men, they are able to combat infections more efficiently, which could help explain the comparable mortality in both groups. The higher rate of acute kidney injury in women despite the lower contrast volume might be explained by the female sex, older age, lower baseline haemoglobin and eGFR, and higher Mehran Score risk for post-PCI contrast nephropathy, which were all shown to predict post-PCI acute kidney injury.^24^, ^25^, ^26^ More incidence of major bleeding in women in the IMPELLA-PL registry is also in accordance with the previous mechanical circulatory support studies.^16^^,^^18^ However, whether female sex is an independent predictor of complications remains disputable, with contemporary evidence suggesting that the increased risk of post-PCI bleeding in women is rather associated with older age, lower hemoglobin, and comorbidities.^27^^,^^28^

### Women undergoing Impella-assisted HR PCI have fewer device-related complications

In contrast to historic studies,^6^ the authors observed fewer device-related complications in women, compared to men. These findings were despite a similar rate of femoral artery access, ultrasound-guided puncture, and contralateral safety access for Impella, and despite a similar rate of radial and femoral access for PCI, along with the median sheath size of 7F in both groups. Surgical access occurred more frequently in women (21.9 % vs. 14.0 %), which might have contributed to more conscious vascular access management, but also to more inflammatory complications. In addition, Impella explantation occurred in the catheterization laboratory for the majority of patients, which likely prevented complications associated with long-term Impella support in both groups.

### Mortality in women undergoing Impella-supported HR PCI

Regarding long-term mortality, the present study is the first to show a trend towards improved 12-month survival in women undergoing Impella-supported HR PCI. The discrepant results between this study and previous studies might be due to differences in baseline characteristics and different follow-up durations. These aspects, along with the potential influence of ethnic or genetic factors on the Impella-supported HR PCI results, should be further explored. Interestingly, similar results were found in a retrospective analysis of 4510 patients with aortic stenosis undergoing isolated surgical aortic valve replacement, where women were older, displayed more non-cardiac comorbidities, and faced a higher operative risk, but had better 5-year survival than men upon adjustment for baseline characteristics.^8^ Still, the observed mortality benefit in the present study should be interpreted with caution, considering the large underrepresentation of women in the registry (12.6 %). The latter is a caveat of all contemporary sex-based subanalyses regarding Impella in the HR-PCI setting, with 26.8 % women in the PROTECT III study, 24.8 % in the Catheter-based Ventricular Assist Devices registry and 22.4 % in the Impella Italian registry.^15^, ^16^, ^17^ Further analyses with larger number of women are crucial to provide statistically relevant and clinically valuable insights into the observed sex differences.

### Clinical implications

The present results support the adoption of Impella during HR-PCI procedures regardless of sex, showing that women experience comparable clinical outcomes from Impella support as men, despite more advanced age and greater complexity of coronary artery disease. This fact should be taken into account during the qualification of patients for Impella-supported HR-CI by the Heart Teams, especially considering the strikingly higher prevalence of Impella-assisted HR-PCI in men. At the same time, the operators and intensivists should be aware of the generally higher risk of complications in women, requiring meticulous preprocedural planning and postprocedural management to ensure the best possible outcomes.

### Strengths and limitations of the study

The present study has multiple strengths, including the multicenter design, the participation of public and mostly academic research centers with the full reimbursement of Impella for the study participants, the standardized way of data collection, the complete follow-up, and the first time ever observed trend towards the improved 12-month survival in women undergoing Impella-supported HR-PCI. It also has several limitations, including the underrepresentation of women, registry-based design limited by the completeness of medical data, lack of an independent event adjudication committee, definition of bleeding complications according to the operator’s judgement, and implantation and removal of Impella during the same procedure in most patients, reflecting the overall clinical stability of the HR-PCI cohort. In addition, the marginal p-values, such as for lower estimated glomerular filtration rate (*p* = 0.053) and fewer device-related complications (*p* = 0.049) in women, should be interpreted cautiously. Importantly, the present results do not refer to the Impella use in the cardiogenic shock setting, since patients with cardiogenic shock were excluded from the analysis due to substantial differences in the baseline characteristics and outcomes between the cardiogenic shock and HR-PCI subpopulations. In addition, the small sample size of the cardiogenic shock patients included in the registry (*n* = 55, including 13-women) would make the gender-subgroup analysis statistically not reliable.^19^

## Conclusions

Women and men undergoing Impella-assisted HR PCI had comparable in-hospital mortality rates, but different complication profiles, with more device-related complications in men and more systemic complications in women. Following hospital discharge, the 12-month survival rate tends to be higher in women, despite their more advanced age and greater complexity of coronary artery disease.

## Funding

This study was funded by a research grant from Abiomed (grant number #69,829,335).

## Authors’ contributions

Conceptualization: AP, AG, JS, TP, MG, JK, Data curation: All authors, Formal Analysis: AP, AG, ML, MK, Funding acquisition: AP, AG, JK, Investigation: All authors, Methodology: AP, AG, ML, MK, Project administration AP, AG, EB, Resources: All authors, Software: AP, AG, ML, ML, Supervision JS, TP, MG, JK, Validation: All authors, Visualization: All authors, Writing-original draft: AP, AG, EB, Writing-review & editing: All authors.

## Permissions

The authors do hereby declare that all Illustrations and Figures in the manuscript are original and do not require reprint permission.

## Data availability

The data underlying this article will be shared on reasonable request to the corresponding author.

## AI statement

No artificial intelligence or machine learning tools were used in the creation, drafting, or editing of this article.

## Declaration of competing interest

Arkadiusz Pietrasik and Aleksandra Gąsecka received a research grant from Abiomed (grant number #69,829,335). Jerzy Sacha is an Impella proctor. Other authors have nothing to disclose.

## Glossary of field-specific terms

Risk-Treatment Paradox: A phenomenon where patients with a higher risk of morbidity and mortality receive less aggressive or guideline-recommended care than lower-risk patients.
Percutaneous Coronary Intervention (PCI): A non-surgical procedure to open narrowed or blocked coronary arteries, often involving stent placement to improve blood flow to the heart.
High-Risk PCI (HR PCI): A PCI procedure performed on patients with complex clinical, anatomical, or procedural challenges, such as severe coronary artery disease, low ejection fraction, or significant comorbidities, which increase the risk of complications.
Mechanical Circulatory Support (MCS): Devices, like Impella, are used to assist the heart’s pumping function, especially during high-risk procedures or heart failure, to maintain stable blood circulation.
Impella: A catheter-based percutaneous device that provides temporary mechanical support to the heart’s left ventricle, used during high-risk PCI to maintain hemodynamic stability.
Cardiogenic Shock (CS): A life-threatening condition where the heart suddenly fails to pump enough blood to meet the body's needs, often requiring emergency mechanical support.
Left Ventricle Ejection Fraction (LVEF): The percentage of blood pumped out of the left ventricle with each heartbeat, used to assess heart function; a lower LVEF indicates weakened heart performance.
Systemic Complications: Adverse events affecting multiple organs or systems, such as infection or kidney injury, which can arise during or after medical procedures.
Device-Related Complications: Adverse events directly associated with the mechanical support device, such as vascular access issues or bleeding.
Major Adverse Cardiac Events (MACE): Serious heart-related complications that can occur during or after treatment, including heart attack, stroke, or need for repeat revascularization.
Mehran Score: A risk assessment tool used to predict the likelihood of contrast-induced nephropathy following PCI, particularly in high-risk patients.
Syntax Score: A scoring system used to assess the severity and complexity of coronary artery disease based on angiography findings.
Multivessel Disease: A condition in which multiple coronary arteries are affected by significant stenosis, increasing the complexity of treatment and procedural risk.
Fractional Flow Reserve (FFR): A technique that measures pressure differences across a coronary artery stenosis to assess the significance of the blockage, guiding the need for intervention.
Extracorporeal Membrane Oxygenation (ECMO): A temporary life-support machine that oxygenates blood outside the body, often used for patients with severe heart or lung failure.
Intra-Aortic Balloon Pump (IABP): A mechanical device inserted into the aorta to improve heart function and blood flow, often used as a bridge during cardiac procedures.
EuroSCORE: A scoring system used to estimate the risk of mortality from cardiac surgery based on patient characteristics and comorbidities.

## References

[1] Pietrasik A., Gąsecka A., Jasińska-Gniadzik K., Szwed P., Grygier M., Pawłowski T. (2023). Roadmap towards an institutional Impella programme for high-risk coronary interventions. ESC Heart Fail.

[2] Van Der Sangen N.M.R., Azzahhafi J., Chan Pin Yin D.R.P.P., Peper J., Rayhi S., Walhout R.J. (2022). External validation of the GRACE risk score and the risk-treatment paradox in patients with acute coronary syndrome. Open Heart.

[3] Saar A., Marandi T., Ainla T., Fischer K., Blöndal M., Eha J. (2018). The risk-treatment paradox in non-ST-elevation myocardial infarction patients according to their estimated GRACE risk. Int J Cardiol.

[4] Mehilli J., King L. (2013). Risk-treatment paradox in women with symptomatic coronary artery disease. Clin Res Cardiol Suppl.

[5] Chieffo A., Buchanan G.L., Mehilli J., Capodanno D., Kunadian V., Petronio A.S. (2018). Percutaneous coronary and structural interventions in women: a position statement from the EAPCI Women Committee. EuroIntervention.

[6] Doyle B.J., Ting H.H., Bell M.R., Lennon R.J., Mathew V., Singh M. (2008). Major femoral bleeding complications after percutaneous coronary intervention patients treated at the Mayo Clinic from 1994 to 2005. JACC Cardiovasc Interv.

[7] Shah T., Haimi I., Yang Y., Gaston S., Taoutel R., Mehta S. (2021). Meta-analysis of gender disparities in In-hospital care and outcomes in patients with ST-segment elevation myocardial infarction. Am J Cardiol.

[8] Peles I., Barrett O., Cafri C., Garcia-Garcia H., Tsaban G., El-Nasasra A. (2023). Predictors of adverse outcome in high-risk percutaneous coronary interventions patients. Can J Cardiol.

[9] Lawton J.S., Tamis-Holland J.E., Bangalore S., Bates E.R., Beckie T.M., Bischoff J.M. (2022). 2021 ACC/AHA/SCAI guideline for coronary artery Revascularization: executive Summary: a report of the American College of Cardiology/American Heart Association Joint Committee on Clinical Practice Guidelines. Circulation.

[10] Chieffo A., Dudek D., Hassager C., Combes A., Gramegna M., Halvorsen S. (2021). Joint EAPCI/ACVC expert consensus document on percutaneous ventricular assist devices. Eur Heart J Acute Cardiovasc Care.

[11] Møller J.E., Engstrøm T., Jensen L.O., Eiskjær H., Mangner N., Polzin A. (2024). Microaxial flow pump or standard care in infarct-related cardiogenic shock. N Engl J Med.

[12] Chitturi K.R., Zhang C., Abusnina W., Sawant V., Banerjee A., Ahmed S. (2025). High-risk percutaneous coronary intervention with or without mechanical circulatory support: will Impella show superiority in the PROTECT IV randomized trial?. Cardiovasc Revasc Med.

[13] Shah T., Lansky A.J., Grines C.L., O’Neill W.W., Moses J.W., Chieffo A. (2022). Mechanical circulatory support in myocardial infarction complicated by cardiogenic shock: impact of sex and timing. J Soc Cardiovasc Angiogr Interv.

[14] Joseph S.M., Brisco M.A., Colvin M., Grady K.L., Walsh M.N., Cook J.L., genVAD Working Group (2016). Women with cardiogenic shock derive greater benefit from early mechanical circulatory support: an update from the cVAD registry. J Interv Cardiol.

[15] Shah T., Abu-Much A., Batchelor W.B., Grines C.L., Baron S.J., Zhou Z. (2023). Sex differences in pLVAD-assisted high-risk percutaneous coronary intervention: insights from the PROTECT III study. JACC Cardiovasc Interv.

[16] Alraies M.C., Kaki A., Kajy M., Blank N., Hasan R., Htun W.W. (2020). Sex-related difference in the use of percutaneous left ventricular assist device in patients undergoing complex high-risk percutaneous coronary intervention: insight from the cVAD registry. Catheter Cardiovasc Interv.

[17] Alessandro Beneduce FZCBCTENGMFDMAMSGTAC (2023). Multicenter registry of patients treated with Impella mechanical circulatory support device in Italy: sex subanalysis. JACC Cardiovasc Interv.

[18] Osman M., Syed M., Abdul Ghaffar Y., Patel B., Abugroun A., Kheiri B. (2021). Gender-based outcomes of impeller pumps percutaneous ventricular assist devices. Catheter Cardiovasc Interv.

[19] Pietrasik A., Gąsecka A., Pawłowski T., Sacha J., Grygier M., Bielawski G. (2023). Multicenter registry of Impella-assisted high-risk percutaneous coronary interventions and cardiogenic shock in Poland (IMPELLA-PL). Kardiol Pol.

[20] Pietrasik A., Gasecka A., Grygier M., Pawlowski T., Sacha J., Kochman J. (2022). Mechanical circulatory support for high-risk percutaneous coronary interventions and cardiogenic shock: rationale and design of the multicenter, investigator-initiated IMPELLA-PL registry. Cardiol J.

[21] Pietrasik P., Gąsecka A., Pawłowski T., Sacha J., Grygier M. (2025). Procedural characteristics and outcomes of patients undergoing Impella-assisted high-risk percutaneous coronary interventions in the IMPELLA-PL registry. Cardiovasc Revasc Med.

[22] Vázquez-Martínez E.R., García-Gómez E., Camacho-Arroyo I., González-Pedrajo B. (2018). Sexual dimorphism in bacterial infections. Biol Sex Differ.

[23] Dias S.P., Brouwer M.C., Van De Beek D. (2022). Sex and gender differences in bacterial infections. Infect Immun.

[24] Lucreziotti S., Centola M., Salerno-Uriarte D., Ponticelli G., Battezzati P.M., Castini D. (2014). Female gender and contrast-induced nephropathy in primary percutaneous intervention for ST-segment elevation myocardial infarction. Int J Cardiol.

[25] Barbieri L., Verdoia M., Nardin M., Marino P., Suryapranata H., De Luca G., Novara Atherosclerosis Study Group (NAS) (2017). Gender difference in the risk of contrast-induced nephropathy in patients undergoing coronary angiography or percutaneous coronary intervention. Angiology.

[26] Rakowski T., Dziewierz A., Węgiel M., Siudak Z., Zasada W., Jąkała J. (2022). Risk factors of contrast-induced nephropathy in patients with acute coronary syndrome. Kardiol Pol.

[27] Spirito A., Gragnano F., Corpataux N., Vaisnora L., Galea R., Svab S. (2021). Sex-based differences in bleeding risk after percutaneous coronary intervention and implications for the academic research consortium high bleeding risk criteria. J Am Heart Assoc.

[28] Mehran R., Chandrasekhar J., Urban P., Lang I.M., Windhoevel U., Spaulding C. (2020). Sex-based outcomes in patients with a high bleeding risk after percutaneous coronary intervention and 1-month dual antiplatelet therapy: a secondary analysis of the LEADERS FREE randomized clinical trial. JAMA Cardiol.

